# The Influence of Interpersonal Trust on Rural Residents' Willingness to Participate in Mutual Aid for the Aged: An Empirical Analysis Based on the Survey Data of Hubei and Henan Provinces

**DOI:** 10.1155/2022/2366425

**Published:** 2022-08-18

**Authors:** Beibei Liu, Yongyong Sun

**Affiliations:** School of Public Administration, Central China Normal University, Wuhan 430000, China

## Abstract

At present, there is a huge gap between supply and demand of old-age services in rural areas of China. Developing rural mutual old-age services is of great significance to remedy the gap. Based on the survey data of 1200 rural residents in Hubei and Henan provinces, this paper adopts binary logistic regression model to analyze the influence of special trust and general trust on rural residents' willingness to participate in mutual care for the aged. The results show that both special trust and general trust have an impact on rural residents' willingness to participate in mutual support for the elderly, but the effect of special trust on rural residents' willingness to participate in mutual support for the elderly is not significant. General trust has a significant promoting effect on rural residents' willingness to participate in mutual care for the aged. Chinese rural residents' trust in village cadres has a significant promoting effect on their willingness to participate in mutual assistance for the aged. The trust of ordinary friends significantly inhibited their willingness to participate. The educational level, living style, and economic status of Chinese rural residents have a positive impact on their willingness to participate in mutual care for the aged. Age, marital status, health status, and intergenerational relationship are inversely correlated with willingness to participate.

## 1. Introduction

According to the seventh national census data, the national population is 1,411.78 million, and the population aged 60 and above is 264.02 million, accounting for 18.70% (among them, the population aged 65 and above is 190.64 million, accounting for 13.50%). Compared with 2010, the proportion of the population aged 60 and above has increased by 5.44 percentage points [1]. The level of rural economic development is backward, the self-security ability of farmers is poor, and they do not have the economic ability and willingness to purchase professional pension services. The national security capacity is relatively inadequate. Although the coverage rate of rural old-age security (including residents' old-age insurance and minimum living security and “five guarantees”) is relatively high on the whole, the level of security is low and it cannot provide adequate old-age security. In addition, both public and private pension institutions in rural areas are lagging behind in development, and it is difficult to provide adequate pension services but also relatively common government failure and market failure phenomenon.

In this case, the development of rural mutual support for the elderly has become an important option to solve the problem. Compared with the development of other forms of old-age security, there are certain comparative advantages in promoting mutual support for the elderly among rural residents. It can not only better meet the rural elderly will not leave home but also fully explore and use the existing rural endowment resources, effectively alleviate the shortage of pension funds, services, talents, and other resources in rural areas, and reduce the pressure of pension service. Since Qiantun Village, Feixiang County, Handan City, Hebei Province, established the rural mutual aid Happiness Home in 2008, Hebei, Gansu, Hubei, Guangxi, and other places have successively carried out the exploration of rural mutual aid old-age care innovation road. From the top-level design aspect, the Central government has incorporated mutual assistance for the elderly into national policies. From the Rural Revitalization Strategy Plan (2018–2020) in 2018 to the “No. 1 Document” of the Central Government, Resolutely Winning the Battle against Poverty in 2020, both emphasize the development of mutual assistance for the elderly in rural areas. From the point of view of the current practice model of mutual assistance for the aged in various places, it can be divided into two main types: one is centralized mutual assistance, where the elderly are gathered together, and the elderly provide each other with pension services and spiritual comfort, such as “rural mutual assistance happy home” and village community care center. The other is diaspora, in which elderly people live scattered at home and participate in mutual care services, such as the “Old Buddy Program” and the “Old Friends Circle”. Although different forms of mutual support for the elderly have achieved certain effects, but only a small scope of fire, unable to start a prairie fire, different mutual support for the elderly are facing an important problem, that is, rural residents have low willingness to participate in mutual support for the elderly. Studies show that only 47.9% of the rural elderly are willing to participate in mutual support for the aged [2], showing the characteristics of low quality, low level, and low willingness of the elderly to participate [3] [4] that rural mutual care for the elderly is not[5]. Some scholars have also found that family size, living style, intergenerational relationship, health status, economic status, and age and marital status in individual characteristic variables have a significant impact on the willingness of rural elderly to support each other for the aged [2, 6, 7]. So far, few scholars have studied the specific impact and effect of interpersonal trust on rural residents' willingness to participate in mutual care for the aged. However, the mutual support for the aged in rural areas being explored in our country is essentially a voluntary social exchange, based on the voluntary cooperation of the elderly in rural areas, so who are the elderly willing to cooperate with the object? Who is not? Who influences older people's willingness to cooperate? How to make use of these influencing factors to enhance the willingness to participate and promote the development of rural mutual care for the elderly? These are the questions to be answered in this paper.

## 2. Theoretical Basis and Research Hypothesis

### 2.1. Interpersonal Trust

Before analyzing the connotation of interpersonal trust, we need to understand the connotation of trust. Some scholars regard trust as an individual behavior [8]. However, more scholars believe that trust is a positive expectation of others' behavior [9–12]. That is, an individual's positive expectation is that others will act in consideration of their own interests. Luhmann [12] divides trust into interpersonal trust and institutional trust. Interpersonal trust is based on the emotional connection established in interpersonal communication, while institutional trust is based on the regulation and restriction of norms, rules, and regulations in interpersonal communication [13]. Barber divides interpersonal trust into general trust and special trust: general trust is an individual's expectation that those who maintain social interaction with him can act according to the role norms, usually the trust of others or strangers in the society; special trust refers to the expectation that the individual can fully shoulder the responsibility and trust of acquaintances, friends, and family members [9]. As a subset of trust, interpersonal trust is a kind of interpersonal relationship in essence. Relationship means that both sides of trust play a role. Therefore, from the perspective of trust objects, many researches divide interpersonal trust into special trust and general trust; special trust is based on acquaintance relationship and general trust facing general social members beyond acquaintance relationship [14, 15]. In short, face-to-face communities, mutual knowledge, and strong social control lead to special trust [16]. General trust is more likely to occur in large communities where strangers or nonacquaintances are the majority [17].

Domestic scholars believe that Chinese trust is significantly different from that of the West [18]. The interpersonal trust in Chinese society results from the inner “love is different” and “people are different”, which is consistent with the “differential pattern” proposed by Fei Xiaotong. In other words, different types of interpersonal trust are individual-centered and gradually weaken from inside to outside with the opening of the intimate distance [19]. Family members, relatives, and friends are the most trusted, followed by the rest of the people, the lowest degree of trust is frequent business contacts, classmates, and most people in society [20]. In this study, interpersonal trust is divided into special trust and universal trust according to the different trust objects of rural residents and the relationship between them. The trust of family members, relatives, and neighbors based on blood relationship and geographical relationship is special trust. The trust of village cadre and other friends is universal trust.

### 2.2. Rural Mutual Assistance for the Elderly

Domestic research on mutual assistance for the elderly is quite abundant. From the perspective of research content, existing research on mutual assistance for the elderly has discussed its connotation, historical evolution, development status, practice mode, participation intention, feasibility, development dilemma, path, and other aspects [5, 21–25]. From the perspective of research, existing studies have been analyzed from the perspective of institutional embeddedness, policy tools, historical anthropology, resocialization, and community governance [26–30]. Combing the existing research results, it can be seen that, on the one hand, the existing research has not paid enough attention to the participants of mutual care for the aged. Rural residents are the participants of rural mutual care for the aged, and their willingness to mutual care for the aged is the premise and basis for the realization of mutual care for the aged. On the other hand, some scholars began to pay attention to the role of trust mechanism in mutual care for the aged, but they focused more on social capital, pointing out that social capital can influence the pension behavior of rural elderly through interaction effect, reciprocity effect, and mutual trust effect [31] Abundant social capital can reduce all kinds of friction in the process of mutual aid and make mutual aid pension can still operate well [32]. Some scholars proposed to build trust mechanism, mutual norms, participation in the network to help the sustainable development of mutual care for the elderly. Trust is the core element of social capital, but few scholars have studied its specific influence and function on mutual pension.

### 2.3. Interpersonal Trust and Willingness to Participate in Mutual Care for the Aged

Rural mutual assistance for the aged is a kind of voluntary mutual assistance and cooperation. In voluntary cooperation, individual behaviors will inevitably affect others, and others' behaviors will also affect individual behaviors, and each individual will influence and interact with each other. These influences will be fed back to their actions of mutual assistance for the aged. Therefore, it is necessary to analyze the influencing factors of participation intention from the dynamic level. It has been proved by literature that trust is a necessary condition for the occurrence of farmers' cooperative behavior, and trust presents the form of differential pattern [34]. The “special trust” based on kinship and quasi-kinship is the action logic of Chinese farmers towards cooperation [35]. Trust is a key factor in promoting mutual pension participation. The cultivation of trust can promote people to reach tacit cooperation and form a good interaction between members of the organization [36]. As a core subset of trust, interpersonal trust affects individual behavior choices through interpersonal communication and interaction in rural residents' daily life and production, thus affecting their willingness to participate in mutual care for the aged.

### 2.4. Research Hypothesis

Based on the above analysis, it can be considered that the interpersonal trust of rural residents may be a key variable affecting the willingness of rural residents to participate in mutual care for the elderly, but the different types of trust it contains may have different effects. With the gradual transformation of Chinese society to modern society, the trust relationship in rural and local society gradually develops from “special trust” to “universal trust”. Based on this, the following research hypotheses are proposed.

  Research hypothesis 1: Rural residents' special trust has a positive impact on their willingness to participate in mutual support for the elderly. The more the rural residents trust their children, relatives, and neighbors, the more inclined they are to participate in mutual support for the aged. On the contrary, they are not inclined to participate in mutual pension.
  Research hypothesis 2: The general trust of rural residents has a positive impact on rural residents' willingness to participate in mutual care for the aged. According to previous studies, there is a “differential order pattern” in the trust structure of Chinese people, and the special trust is much higher than the general trust. Therefore, hypothesis 2 can be divided into: the higher the trust degree of rural residents to other friends and village cadres, the higher their willingness to participate in mutual pension; otherwise, they are less willing to participate in mutual pension.
  Research hypothesis 3: Special trust has no significant impact on rural residents' willingness to participate in mutual care for the elderly, while general trust has a significant impact.

## 3. Data Source and Model Design

### 3.1. Data Sources

The research data are from self-tuning data of the research group in 2019. In July 2019, members of the research group took Zhijiang City of Hubei Province and Dengzhou City of Henan Province as the basic sampling framework, and adopted random sampling method to select a number of villages in each place to conduct a questionnaire survey. The purpose of the survey is to investigate the situation of rural residents' mutual support for the elderly, and the respondents are mostly middle-aged and elderly people in rural areas. The content of the questionnaire mainly includes three parts: personal basic information, social network, and the current situation of rural mutual support for the aged. The measure items in the questionnaire are all from the integration of existing literature, and some of the research measures are from western literature, and the method of back-translation is used to ensure the accuracy of the description of measure questions in the questionnaire. The questionnaire survey was completed by one-to-one structured questioning. 1500 questionnaires were distributed, 1200 were recovered, and 1056 valid questionnaires were obtained after eliminating invalid ones. A preliminary survey was conducted before the formal investigation to ensure the reliability and validity of the questionnaire.

### 3.2. Model  Selection

In this paper, the willingness to support the elderly is a binary variable, that is, willing and unwilling. Therefore, this paper selects binary logistic regression model. The model is set as follows:

(1) $$\begin{array}{c} y_{i}=x_{i}^{\prime }\beta +\varepsilon_{i}, \end{array}$$

(2) $$\begin{array}{c} P\left(y=1|x\right)=F\left(x,\beta \right), \end{array}$$

(3) $$\begin{array}{c} P\left(y=1|x\right)=F\left(,\beta \right),=\wedge \left(x^{\prime },\beta \right)=\frac{\exp\left(x^{\prime },\beta \right)}{1+\exp\left(x^{\prime },\beta \right)}, \end{array}$$

(4) $$\begin{array}{c} f\left(y_{i}|x_{i},\beta \right)={\left[\wedge \left(x_{i}^{\prime },\beta \right)\right]}^{y_{i}}\left[1-\wedge {\left(x_{i}^{\prime },\beta \right)}^{1-y_{i}}\right], \end{array}$$

(5) $$\begin{array}{c} \ln\;f\left(y_{i}|x_{i},\beta \right)=y_{i}\ln\left[\wedge \left(x_{i}^{\prime },\beta \right)\right]+\left(1-y_{i}\right)\ln\left[1-\wedge \left(x_{i}^{\prime },\beta \right)\right], \end{array}$$

In the model, *y*_*i*_ is the dependent variable, *X*_*i*_ is the independent variable, *β* is the coefficient, and *ε* is the random disturbance term. Combined with this study, the final model is:

(6) $$\begin{array}{c} \log\;itP\left({\text{willingness}}_{i}=1|\text{special}{\text{ trust}}_{i},\text{universal}{\text{ trust}}_{i},{\text{controls}}_{i}\right)\\ =\alpha_{i}\text{special}{\text{ trust}}_{i}+\beta_{i}\text{universal}{\text{ trust}}_{i}+\chi {\text{controls}}_{i}, \end{array}$$

where subscript *I* represents the *i*th rural resident to be interviewed. The explained variable is a 0–1 binary variable about the willingness of rural residents to participate in the mutual support for the elderly. If rural residents are willing to participate in the mutual support for the elderly, the value is 1; otherwise, it is 0. Special trust_*i*_ represents the special trust of *I* rural residents interviewed, and universal trust_*i*_ represents the general trust of the ith rural residents surveyed. Controls_i_ represents the control variable of the ith rural resident.

In this paper, IBM SPSS26.0 software is used to analyze variables at different levels. Model 1 only adds control variables for regression. Model 2 puts three variables of special trust on top of Model 1. Model 3 puts two variables of general trust based on Model 1. Model 4 puts all the variables for control variables, general trust, and special trust.

### 3.3. Variable Definition

According to the above theoretical analysis and research assumptions, variables are defined as follows.

The dependent variable is the willingness of rural residents to participate in mutual care for the aged, and the index used to measure it comes from the survey question “Are you willing to provide mutual care for the aged? The answer is designed with two choices, i.e., willing = 1 and unwilling = 0, which is a dichotomous-dependent variable.

The independent variables include special trust and universal trust. In the aspect of special trust, three variables are set up: trust to children, trust to relatives, and trust to neighbors. In the specific investigation process, considering the understanding and acceptance ability of rural residents, the above three variables were transformed into the following questions: “I think children: most of them are not trustworthy, most of them are not trustworthy, the trustworthy and untrustworthy are evenly divided, most of them are trustworthy, most of them are trustworthy” “I think relatives: “I think neighbors: Majority untrustworthy, Majority untrustworthy, Majority untrustworthy, Majority untrustworthy, majority trustworthy, majority trustworthy.” In terms of general trust, two variables are set: trust to village cadres and trust to other friends. These two variables were translated into the following question: “I think village cadres: the vast majority are not trusted, the majority are not trusted, the trustworthy and untrustworthy are evenly divided, the majority are trusted, the vast majority are trusted.” “I think other friends: most untrustworthy, most untrustworthy, trustworthy and untrustworthy, most trustworthy, most trustworthy.” “Most untrustworthy, most untrustworthy, trustworthy and untrustworthy half, most trustworthy, most trustworthy” reflects the trust intensity and is assigned 1, 2, 3, 4, 5, respectively.

Control variables. According to the existing research conclusions, education level, family size, living style, intergenerational relationship, health status, economic status, age, and marital status among individual characteristic variables have a significant impact on the willingness of rural elderly to support each other for the aged [2, 6, 7]. In order to avoid research bias caused by omission of variables, other explanatory variables are controlled in this study, which are mainly divided into personal characteristics and family characteristics. One is personal characteristic variables, which mainly include gender, age, marital status, education level, and health status. Where, gender, assign “male” to 1 and “female” to 0; age is assigned according to different age groups; marital status was set as a categorical variable for four conditions in the questionnaire: unmarried, spousal, spousal (widowed), and spousal (divorced); education level, without any education is assigned 1, primary school is assigned 2, junior high school is assigned 3, senior high school is assigned 4, technical secondary school is assigned 5, and college or above is assigned 6; health status is the self-rated health status of the interviewees, and different health levels are assigned different values. The second is family characteristic variables, including intergenerational relationship, living style, and economic status. Where the intergenerational variables were set as frequency of visits by adult children, with a value of 1 per week, 2 for half a month, 3 for one month, 4 for several months (from February to May), 5 for half a year, and 6 for one year or more. Residence mode is set as a classification variable for different situations. Economic status is set as personal income and is a continuous variable(Table 1).

## 4. Data Analysis and Research Findings

### 4.1. Descriptive Statistics of the Current Situation of Rural Mutual Assistance for the Elderly

Among the rural residents surveyed, 49.6 percent were male and 50.3 percent were female. Most of them had education below primary school, accounting for 53.7%. The age was 50–69 years old, accounting for 55.5%. Most of them were married, accounting for 83.5%; The health status was mainly relatively healthy and very healthy, accounting for 59.3%. The main source of monthly income is labor income, accounting for 62.4%. The majority of residents lived with their spouses or their children, accounting for 70.4% (see Table 2).

**Figure 1:**
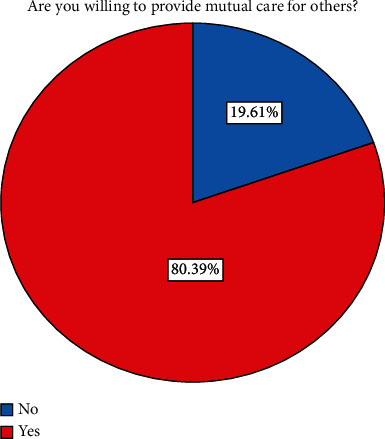
Willingness to participate in mutual pension.

Generally speaking, rural residents have a higher willingness to participate in mutual support for the elderly. To the question “Are you willing to provide mutual care for others?” When answering this question, 80.39% of respondents are willing to provide mutual care for the aged. Only 19.61% of the respondents answered that they are not willing to provide mutual care for the aged (see Figure 1).

In terms of gender, there is a certain difference between male and female rural residents' willingness to participate in mutual care for the aged. The willingness of female rural residents to participate in mutual care for the elderly was higher than that of male rural residents, and the willingness of female rural residents to participate in mutual care for the elderly reached 79.37%. In terms of age, rural residents of different age groups have different willingness to participate in mutual assistance for the elderly, showing the characteristics of younger people with higher willingness to participate and older people with lower willingness to participate. The willingness of rural residents under 70 years old to participate in mutual care for the elderly is higher, all above 80% level, and the willingness of rural residents under 50 years old to participate in mutual care for the elderly is the highest. With the increase of age, the willingness to participate tends to decrease, and the willingness to participate of rural residents of all ages over 70 is around 60%. In terms of physical health, people with better physical health have higher willingness to participate, while those with poor physical health have lower willingness to participate. The rural residents of mutual endowment to participate are above the 80% level, health of rural residents' mutual endowment to participate reflected to participate in the transition between the good and bad health, very unhealthy and less healthy rural households mutual endowment to participate are around 65% level. In terms of marital status, there are differences among different marital status. Rural residents in the unmarried state are generally more willing to participate in mutual support for the elderly. In the married state, it is divided into different situations. Rural residents with spouse have a higher willingness to participate in mutual care for the aged, reaching 79.86%, while rural residents without spouse have a lower willingness to participate in mutual care for the aged, among which the widowed rural residents have the lowest willingness to participate in mutual care for the aged (see Table 3).

**Figure 2:**
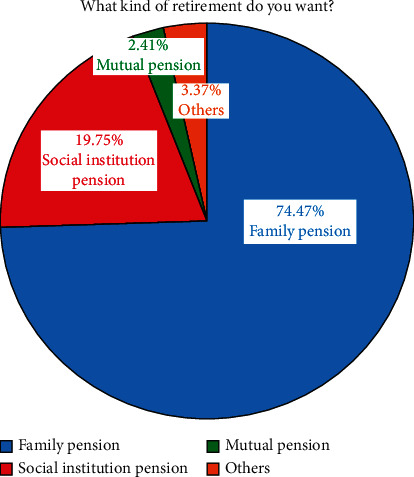
The choice of pension.

Most elderly people in rural China want to spend their old age at home, and their favorite way to provide for the aged is family care. 74.47% of respondents hope to choose family pension, 19.75% social institution pension (nursing home), 2.41% mutual support pension, and 3.37% other options (see Figure 2).

**Figure 3:**
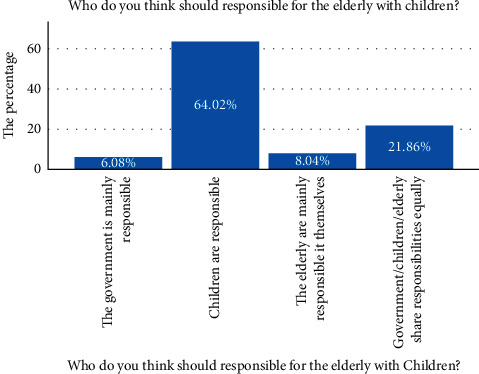
Elderly care responsibilities.

Since ancient times, there has been a concept of “raising children for old-age” in China. For most of the elderly in China, the main responsibility for supporting their children is their children. The findings of this study confirm this, 64.02 percent of the respondents said that children should be responsible for the elderly with children, followed by 8.05 percent who chose the elderly themselves, 21.86 percent who chose the government, their children, and the elderly, and 6.08 percent who chose the government (see Figure 3).

**Figure 4:**
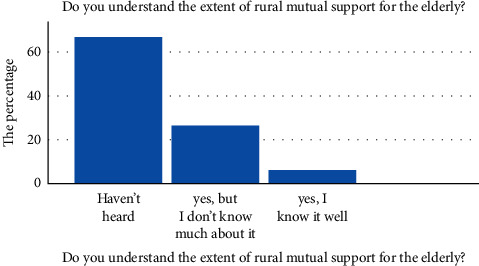
Understanding of mutual pension.

With the aggravation of China's aging population, the family's supporting function will gradually weaken. To solve the pension problem, we need to fundamentally change the traditional pension concept. However, according to the survey of this study, elderly people in rural China have a low awareness of the mutual assistance pension model. 66.9% of respondents have not heard of mutual support for the elderly, 26.6% of respondents have heard of it but do not know much about it, and only 6.4% of respondents have heard of it very well (see Figure 4).

**Figure 5:**
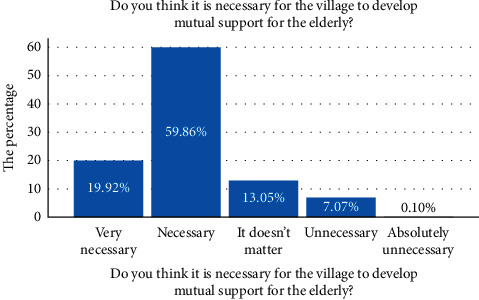
The necessity of developing the model of mutual support for the aged.

In China's rural areas, with the aging of the population and the large-scale outflow of young and middle-aged labor force, the demand for elderly care services is increasing, and many elderly people have realized the necessity of mutual support for the elderly model. The survey of this study shows that 59.86 percent of respondents believe that it is necessary to develop mutual support for the elderly in the village, and people have recognized the necessity of mutual support for the elderly (see Figure 5).

### 4.2. Descriptive Statistics of Independent Variables and Control Variables

The core explanatory variables in this paper include special trust and universal trust element. Special trust mainly includes three variables: trust degree to children, trust degree to relatives, and trust degree to neighbors. General trust includes two variables: trust in village cadres and trust in other friends. Sex, age, education, marital status, and residence status were used as control variables. As shown in Table 3, in terms of the mean values of each variable, the mean values of the three variables in special trust are higher than those of the two variables in general trust. Among them, the mean value of trust in children is the largest, while that of other friends is the lowest. It shows that the current trust pattern of farmers based on family is obvious, and the interpersonal trust of farmers shows an obvious differential order pattern from close to sparse, from inside to outside. This is consistent with the traditional differential pattern theory and shows the family-oriented characteristics of traditional Chinese rural society summarized by previous researches. The difference is that the average trust degree of village cadres is higher than that of other friends. Since the reform and opening up, rural grassroots democracy construction has made certain achievements. Village cadres play a very important role in rural governance, and the average trust degree of farmers on village cadres is higher than that of other friends.

### 4.3. Multicollinearity Test

Before the regression analysis, considering that there may be internal correlations among variables such as rural residents' trust in children, relatives, neighbors, village cadres, and other friends, this paper makes a multicollinearity diagnosis for each variable. Generally, when VIF > 3, there is a certain degree of multicollinearity between the respective variables. When VIF > 10, there is a high degree of collinearity between the respective variables. By selecting trust in children as the dependent variable and the remaining variables as the independent variables, the estimated results of collinearity test are shown in Table 3. VIF is less than 10. Based on all the estimated results, the collinearity correlation degree between the respective variables is within a reasonable range (Table 4).

### 4.4. Binary Logistic Regression Results

In order to explain the effect of general trust and special trust on the willingness of rural residents to participate in mutual support for the aged, the regression results are shown in Table 5; three variables of special trust were added in model 2, two variables of general trust were added in model 3, and special trust and general trust were added in model 4. As the final model, the observed value of the Hosmer-Lemeshow statistic was 6.512, and the probability *P* was. 590 and is higher than significant level *α*, so the null hypothesis should not be rejected. It is considered that there is no significant difference between the distribution derived from the actual value of the sample and that derived from the predicted value, and the goodness of fit of the model is better. From model 1 to model 4, Nagelkerke *r*^2^ increased from 0.087 to 118, an increase of 35.632%. This shows that interpersonal trust plays an important role in rural residents' willingness to participate in mutual support for the aged (Table 6).

1. From model 2 and model 3, the two regression results show that trust to children and trust to neighbors have a positive effect on the willingness of rural residents to participate in mutual support for the aged, that is to say, the higher the degree of trust to children and neighbors, the higher the willingness of mutual support and participation. The trust to relatives has a negative effect on the participation intention. The higher the trust degree to relatives, the lower the participation intention. The results support hypothesis 1 that the higher the trust level of rural residents towards their children and neighbors, the more likely they are to participate in mutual support for the aged, and vice versa, the less likely they are to participate in mutual support for the aged. However, the variable of trust in relatives has a negative effect on the willingness of rural residents to participate in mutual support for the aged. The possible explanation is that under the background of the current urbanization process in China, a large number of rural young and strong laborers have left their hometown and the outflow of rural labor forces blocks or limits the effectiveness of support. The old people in rural areas cannot rely on their children for their old-age support, while the relatives formed by blood relationship and geography can provide support to the old people nearby. When there is a need for old-age support, the old people can seek help from relatives; therefore, it is not a priority to participate in mutual support for the aged, so the trust of relatives has a negative effect on this variable.
2. The regression results of model 3 and model 4 show that in interpersonal trust, the two variables of universal trust have a significant effect on rural residents' willingness to participate in mutual support for the aged and have passed the significance test (*P* < 0.001). In the final model 4, the higher the trust of rural residents to the village cadres, the higher their willingness to participate in mutual support for the aged. Village cadres are grassroots organization managers such as village party secretaries and village committee directors who control the village's formal power resources. They are the village's elite; village elites play an important role in the modernization of Chinese villages. In the mutual support service for the aged, the higher the rural residents' trust to the village cadres, the higher their willingness to participate, which is consistent with the scholars' theory of elite governance. Trust to other friends and mutual support participation intention showed the opposite effect, the lower the trust to other friends, the higher the mutual support participation intention. First, the trust of other friends has an important impact on the willingness of rural residents to provide for the elderly, we should pay full attention to this factor. Secoond, the Rural Society in China is an acquaintance society, in which the active subjects have enough understanding of each other due to long-term contact, and the degree of Information asymmetry interaction is low, as a result, rural residents are more willing to choose acquaintances as cooperative partners to reduce the risk of old-age care. Therefore, the more they distrust other friends, they are more willing to choose mutual assistance based on acquaintances, the more willing you are to participate.
3. The special trust variable had no significant effect on the willingness to participate (*P* > 0.05), but the general trust variable had a significant effect on the willingness to participate (*P* < 0.001). Hypothesis 3 of this paper is demonstrated. Special trust has no significant effect on rural residents' willingness to participate in mutual care for the aged, while general trust has a significant effect. In today's rural society, the interpersonal trust pattern of rural residents is changing: before the traditional blood relationship as the core of relative closeness which is formed by the special trust relationship with the stability of the society is gradually changing, special trust occupying the leading status in the pattern of relationships is changing, the situation of interpersonal trust relationship gradually by the “special trust” the direction of “universal trust”; therefore, special trust has no significant effect on rural residents' willingness to participate in mutual care for the aged, while general trust has a significant effect.
4. Among the control factors, the controlling factors, gender, education level, living style, and economic status have a positive effect on the willingness to participate in mutual care for the elderly. In particular, educational level has a significant impact on willingness to participate. The higher the culture, the higher the willingness to participate. The four variables of age, marital status, health status, and intergenerational relationship have a reverse effect on rural residents' willingness to participate in mutual care for the aged. The younger the age, the higher the willingness to participate. Older people who live alone are more likely to participate in mutual care. Rural residents with poor health status had higher willingness to participate. The better the intergenerational relationship, the higher the frequency of home visits of adult children, the lower their willingness to participate. The possible explanation is that the elderly living alone and in poor health has greater demand for pension services, and their willingness to participate is higher.

## 5. Conclusion and Discussion

Based on the above analysis, the main conclusion of this paper can be summarized as follows. (1) Both special trust and general trust have an impact on rural residents' willingness to participate in mutual care for the aged, but the impact of special trust on rural residents' willingness to participate in mutual care for the aged is not significant. General trust has a significant promoting effect on rural residents' willingness to participate in mutual care for the aged. (2) Chinese rural residents' trust in village cadres has a significant promoting effect on their willingness to participate in mutual assistance for the elderly. The trust of ordinary friends significantly inhibited their willingness to participate. (3) Gender, education level, living style and economic status of Rural Chinese residents have a positive impact on their willingness to participate in mutual care for the elderly; In particular, the degree of education has a significant promoting effect on the willingness to participate, the higher the degree of education of the elderly, the higher the willingness to participate in mutual support for the elderly. (4) There is an inverse correlation between age, marital status, health status, intergenerational relationship and rural residents' willingness to participate in mutual care for the aged. The older people who live alone have poor health, poor intergenerational relationship with their children, and higher willingness to participate in mutual support for the aged.

It should be pointed out that the willingness of rural residents to participate in mutual care for the aged will be affected by multidimensional factors. This study only analyzes from the perspective of interpersonal trust, and a more systematic investigation can be carried out by combining macrolevel factors with microlevel factors. In addition, the sample of this study is a representative sample of Chinese rural residents, and the research conclusion is only applicable to Chinese rural residents. In future studies, we still need to further expand the sample range and improve the external validity and robustness of research conclusions.

## Figures and Tables

**Table 1:** Variable description table.

The variable name		Variable assignment
The dependent variable		
Willingness	Are you willing to provide mutual care for others?	1 = yes 2 = no

The independent variables		
Special trust	1. Trust in children	1 = The vast majority are not credible
	2. Trust in your neighbor	2 = Most cannot be trusted
	3. Trust in relatives	3 = The trustworthy and the untrustworthy are half and half

General trust	1. Trust in village cadres	4 = Most trusted
	2. Trust in other friends	5 = Overwhelmingly credible

Control variables		
Gender	1 = male 0 = female	
Age	1 = under 50 2 = 50–59 3 = 60–69 4 = 70–79 5 = 80–89 6 = More than 90	
Degree of education	1 = unlettered 2 = primary school 3 = Junior high school 4 = senior high school 5 = secondary technical school 6 = above	
Marital status	1 = unmarried 2 = have a spouse 3 = death of a spouse 4 = divorced	
Health condition	1 = very health 2 = a healthier 3 = general 4 = less healthy 5 = It's not healthy	
Personal annual income	1 = under 5000 CNY 2 = 5000≤ *x* < 10000 CNY 3 = 10000 ≤ *x* < 20000 CNY 4 = 20000 ≤ *x* < 30000 CNY 5 = 30000 ≤ *x* < 40000 CNY 6 = More than 40000 CNY	
Living pattern	1 = live alone 2 = with spouse 3 = with children 4 = with spouse and children 5 = with relatives	
Intergenerational relationship	Frequency of visits by adult children: 1 = every day 2 = half a month 3 = a month 4 = a few months 5 = half a year 6 = one year and above	

**Table 2:** Basic information of the survey sample.

Project	Category	Percentage
Gender	Male	49.6
	Female	50.3

Degree of education	Unlettered	20.8
	Primary school	32.9
	Junior high school	27.5
	Senior high school	13
	Secondary technical school	2.5
	Above	3.3

Health condition	Very health	17.5
	a healthier	41.8
	General	24
	Less healthy	13.7
	It's not healthy	3

Marital status	Unmarried	2.3
	Have a spouse	83.5
	Death of a spouse	12.9
	Divorced	1.3

Age	Under 50	23.1
	50–59	29.5
	60–69.	26
	70–79.	17.4
	80–89.	3.6
	More than 90	0.5

Living pattern	Live alone	8.7
	With spouse	39.5
	With children spouse, children	18.3
	With relatives	30.9
		2.6

**Table 3:** Cross analysis of basic characteristic variables and willingness to participate in mutual pension.

Variable	Variable classification	Willingness to participate in mutual pension			
		Yes		No	
		Number	Percentage	Number	Percentage
Gender	Male	393	76.46	121	23.54
	Female	427	79.37	111	20.63

Age	≤50	206	85.12	36	14.88
	50–59	251	81.23	58	18.77
	60–69	219	80.22	54	19.78
	70–79	117	64.29	65	35.71
	80–89	22	57.89	16	42.11
	≥90	3	60.00	2	40.00

Marital status	Unmarried	19	79.17	5	20.83
	Have a spouse	694	79.86	175	20.14
	Death of a spouse	90	66.67	45	33.33
	Divorced	10	71.43	4	28.57

Health condition	Very healthy	155	84.70	28	15.30
	Healthier	360	82.19	78	17.81
	General	192	76.49	59	23.51
	Less healthy	90	62.50	54	37.50
	Not healthy	21	65.63	11	34.38

**Table 4:** Multicollinearity diagnosis.

	Nonstandardized coefficient		The standard coefficient			Collinearity statistics	
	B	Standard error of	A trial version	*t*	Sig.	Tolerance	VIF
(Constant)	2.765	0.119		23.143	0.000		
Trust in village cadres	0.077	0.028	0.094	2.741	0.006	0.643	1.556
Trust in neighbor	0.073	0.049	0.071	1.485	0.138	0.329	3.039
Trust in relatives	0.354	0.045	0.363	7.919	0.000	0.361	2.772
Trust in other friends	−0.023	0.030	−0.029	−0.761	0.447	0.528	1.895

A: dependent variable: trust degree of children							

**Table 5:** Descriptive statistics of independent and control variables.

		Minimum	Maximum	Mean	Mean standard error
Special trust	Trust in children	1	5	4.67	0.023
	Trust in relatives	1	5	4.00	0.024
	Trust in your neighbor	1	5	3.91	0.023

General trust	Trust in village cadres	1	5	3.70	0.028
	Trust in other friends	1	5	3.53	0.029

Control variable	Gender	1	2	1.50	0.016
	Age	1	6	2.50	0.036
	Degree of education	1	6	2.54	0.038
	Marital status	1	4	2.13	0.013
	Health condition	1	9	2.43	0.032
	Personal annual income	1	6	2.52	0.044
	Living pattern	0	6	2.78	0.034
	Intergenerational relationship	1	6	4.07	0.074

**Table 6:** Regression results.

Change the amount	Model 1	Model 2	Model 3	Model 4
Trust in children		0.114		0.081
		(0.163)		(0.165)

Trust in relatives		−0.518		−0.440
		(0.277)		(0.246)

Trust in your neighbor		0.270		0.260
		(0.231)		(0.262)

Trust in village cadres			0.273^∗∗∗^	0.287^∗∗∗^
			(0.133)	(0.143)

Trust in other friends			−0.353^∗∗∗^	−0.273^∗∗∗^
			(0.132)	(0.166)

Gender	0.219	0.209	0.199	0.196
	(0.215)	(0.217)	(0.219)	(0.220)

Age	−0.091	−0.092	−0.123	−0.123
	(0.110)	(0.111)	(0.112)	(0.1138)

Level of education	0.279^∗∗^	0.244^∗∗^	0.223^∗∗^	0.205^∗∗^
	(0.124)	(0.126)	(0.125)	(0.127)

Marital status	−0.103	−0.157	−0.103	−0.146
	(0.247)	(0.249)	(0.249)	(0.251)

Health condition	−0.242^∗∗^	−0.254^∗∗^	−0.232^∗∗^	−0.235^∗∗^
	(0.111)	(0.113)	(0.113)	(0.114)

Personal annual income	0.090	0.078	0.084	0.076
	(0.096)	(0.097)	(0.098)	(0.099)

Living pattern	0.108	0.116	0.102	0.103
	(0.103)	(0.104)	(0.104)	(0.105)

Intergenerational relationship	−0.43	−0.053	−0.053	−0.060
	(0.053)	(0.054)	(0.054)	(0.055)

Constant term	1.211	2.004	1.806	2.031
	(1.012)	(1.371)	(1.175)	(1.379)

Chi-square	40.515	46.241	40.695	52.813
	(*P*=0.001)	(*P*=0.001)	(*p* ≤ 0.001)	(*P* ≤ 0.001)

−2 logarithmic likelihood	628.249	622.065	612.697	609.128
Nagelkerke *R*^2^	0.093	0.106	0.115	0.122

*Note*. Standard error in parentheses, standard regression coefficient outside parentheses; ^∗^,^∗∗^, and ^∗∗∗^ were significant at the level of 0.05, 0.01, and 0.001, respectively.

## Data Availability

The datasets used and analyzed during the current study are available from the corresponding author upon reasonable request.
